# The complete mitogenome of Sokolov’s Dwarf Hamster (*Cricetulus sokolovi*) and implication of phylogenetic status

**DOI:** 10.1080/23802359.2021.1914212

**Published:** 2021-07-06

**Authors:** Boxin Qin, Fei Xie, Dan Chen, Qiong Wang, Shunde Chen

**Affiliations:** College of Life Sciences, Sichuan Normal University, Chengdu, China

**Keywords:** Cricetinae, mitogenome structure, phylogeny

## Abstract

There is still an obvious lack of information on Sokolov's Dwarf Hamster (*Cricetulus sokolovi*) which belongs to subfamily Cricetinae because the species is only rarely found in Gobi desert. In this study, we obtained the complete mitochondrial genome sequences of *C. sokolovi.* The genome is 16,292bp in length and has a base composition of 33.5% A, 30.5% T, 22.9% C, and 13.1% G. The mitogenome structure, consisting of 13 protein-coding genes, two rRNA genes (12S rRNA and 16S rRNA), 22 tRNA genes, and one control region, is similar to that of typical vertebrate mitochondrial genomes of other rodents. We restructured a Bayesian phylogenetic tree by using 12 species belonging to subfamily Cricetinae. As indicated by the phylogenetic tree, genus *Cricetulus* is polyphyletic group, and *C. Sokolovi* is the closest relative of *Cricetulus griseus*. The mitochondrial genome can provide basic data for further study on the phylogenetic relationship of subfamily Cricetinae.

The type locality of *Cricetulus sokolovi* (Orlov and Malygin 1988) belonging to subfamily Cricetinae is Bayanhong Lake, southwest bank of Orog Lake, western Mongolia. Distributed in a relatively narrow geographical range, Sokolov's Dwarf Hamster can only be found in western and southern Mongolia, Inner Mongolia Autonomous Region and Gansu province of China (Lunde et al. 2008; Zhao et al. 2016). Previously the species was regarded as a subspecies of *Cricetulus barabensis,* but now both the chromosome and molecular data provide strong evidence for the species status of Sokolov’s hamster (Poplavskaya et al. 2017). In this study, we reported the complete mitochondrial genome of *C. sokolovi*, and reexamined its phylogenetic relationships within Cricetinae.

This hamster was captured in Sonid Right Banne, Inner Mongolia Autonomous Region at an altitude of 1138 m (Latitude: 42.624089°N; Longitude: 112.618343°E). The specimen of *C. sokolovi* was kept in Sichuan Academy of Forestry, Chengdu (Shaoying Liu, email: shaoyliu@163.com) under the voucher number SAF19114. Total genomic DNA was extracted from the specimen tissue by TRIzol^®^ Reagent, which has been deposited at the College of Life Sciences, Sichuan Normal University, Chengdu. The library was constructed by nano DNA sample prep Kit. The mitogenome was sequenced using Illumina Hiseq 4000 sequencing platform and assembled using SPAdes v3.10.1 (Nurk et al. 2013) and GapCloser v1.12 (Luo et al. 2012). The complete mitochondrial genome was annotated using MITOS (Bernt et al. 2013).

The length of complete mitochondrial genome sequence of *C. sokolovi* was 16,292 bp. The organization and order of the genome sequence were similar to that of other rodents’ mitochondrial genomes, which are composed of 13 protein-coding genes, two rRNA genes, 22 tRNA genes, and one control region. Most mitochondrial genes were encoded on the H-strand, except for the ND6 gene and eight tRNA genes (tRNA^Gln^, tRNA^Ala^, tRNA^Asn^, tRNA^Cys^, tRNA^Tyr^, tRNA^Ser^, tRNA^Pro^, and tRNA^Glu^). Several overlaps were identified between different protein-coding genes as shown in ATP8-ATP6, ATP6-COX3, ND4L-ND4, and ND5-ND6. The base composition of complete mitochondrial genome is 33.5% A, 30.5% T, 22.9% C, and 13.1% G. This obviously reflects the typical A–T rich pattern seen in the vertebrate mitochondrial genome (Partridge et al. 2007).

Thirteen concatenated mitochondrial protein genes from *C. sokolovi* and other 11 rodent mitogenomes were utilized to perform phylogenetic analysis through Bayesian inference (BI), and BEAST V1.6.1 was used for this process (Drummond et al. 2012). *Eothenomys melanogaster* and *Eothenomys miletus* were selected as outgroups. The details of BI analysis methods were consistent with those in previous study (Chen et al. 2020), and the best-fit GTR + I + G model of DNA substitution was selected using Akaike Information Criterion (AIC) test in JModelTest 2 (Darriba et al. 2012). As indicated by the phylogenetic tree (Figure 1), *C. sokolovi* is sister to *C. griseus* and the genus *Cricetulus* is polyphyletic. *Cricetulus migratorius* does not cluster with other *Cricetulus* but stands as a sister branch to a well-supported grouping of *Cricetus + Allocricetulus.* Besides, *C. kamensis* clusters together with *Phodopus roborovskii* and *Phodopus sungorus*, suggesting that there is a close phylogenetic relationship between them. Nowadays, the classification of subfamily Cricetinae is still controversial. As a result, in order to gain some better insights into the phylogenetic relationship within Cricetinae, more complete mitochondrial genome sequences are needed.

**Figure 1.**
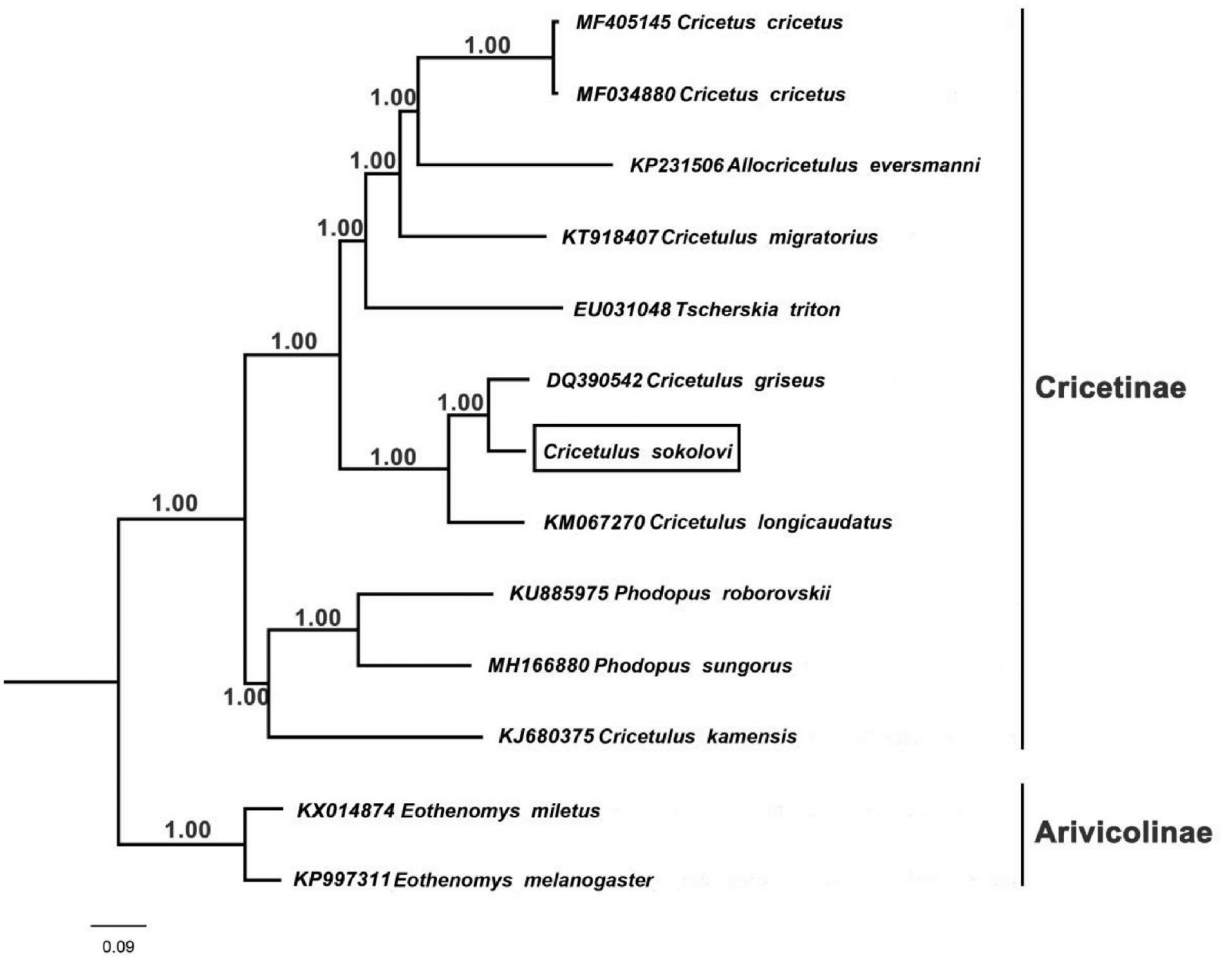
Bayesian phylogenetic tree based on 13 protein genes of mitochondrial genome. Numbers by the nodes indicate Bayesian posterior probabilities.

## Data Availability

Mitogenome data supporting this study are openly available in GenBank at nucleotide database, https://www.ncbi.nlm.nih.gov/nuccore/MW114661. Associated BioProject, https://www.ncbi.nlm.nih.gov/bioproject/PRJNA694342. BioSample accession number at https://www.ncbi.nlm.nih.gov/biosample/SAMN17517416 and Sequence Read Archive at https://www.ncbi.nlm.nih.gov/sra/SRR13517931.
